# WHODAS 2.0 Can Predict Institutionalization among Patients with Traumatic Brain Injury

**DOI:** 10.3390/ijerph16091484

**Published:** 2019-04-26

**Authors:** Shih-Wei Huang, Kwang-Hwa Chang, Reuben Escorpizo, Feng-Hang Chang, Tsan-Hon Liou

**Affiliations:** 1 Department of Physical Medicine and Rehabilitation, Shuang Ho Hospital, Taipei Medical University, Taipei 23561, Taiwan; 13001@s.tmu.edu.tw; 2 Department of Physical Medicine and Rehabilitation, School of Medicine, College of Medicine, Taipei Medical University, Taipei 11031, Taiwan; 3 Graduate Institute of Injury Prevention and Control, College of Public Health, Taipei Medical University, Taipei 11031, Taiwan; chang2773@gmail.com (K.-H.C.); fhchang@tmu.edu.tw (F.-H.C.); 4 Department of Physical Medicine and Rehabilitation, Wan Fang Hospital, Taipei Medical University, Taipei 11696, Taiwan; 5 Department of Rehabilitation and Movement Science, College of Nursing and Health Sciences, University of Vermont, Burlington, VT 05401, USA; escorpizo.reuben@gmail.com; 6 Swiss Paraplegic Research, 6207 Nottwil, Switzerland

**Keywords:** traumatic brain injury (TBI), institutionalization, predictor, International Classification of Functioning, Disability, and Health, Taiwan, World Health Organization Disability Assessment Schedule 2.0

## Abstract

Patients with traumatic brain injury (TBI) often present with disabilities associated with a high burden of care for caregivers or family members at home. When family members cannot afford to care for patients with TBI, they are often required to find them residence in long-term care institutions. To date, there are no quantitative assessment tools developed to predict institutionalization. Therefore, this study analyzed the accuracy of the World Health Organization Disability Assessment Schedule 2.0 (WHODAS 2.0) for predicting the institutionalization of patients with TBI. We designed a cross-sectional study using a nationwide disability database. We analyzed the data of 8630 patients with TBI with injury for more than six months from the Taiwan Data Bank of Persons with Disability during July 2012–October 2018. The demographic data and WHODAS 2.0 standardized scores of patients with TBI who resided in community and long-term care institutions were analyzed. Receiver operating characteristic curve (ROC) analysis was performed to investigate the predictive accuracy of WHODAS 2.0 for being institutionalized, and the optimal cut-off point was determined using the Youden index. Binary logistic regression was employed to determine the predictors of the participants being institutionalized. The WHODAS 2.0 scores in each domain were lower in the community group than in the institutionalized group. ROC analysis revealed the highest accuracy for the summary scores of WHODAS 2.0 (area under the curve = 0.769). Binary logistic regression revealed that age, gender, work status, urbanization level, socioeconomic status, severity of impairment, and WHODAS 2.0 domain scores were factors associated with the institutionalization status of patients with TBI. Our results suggest that WHODAS 2.0 may be a feasible assessment tool for predicting the institutionalization of patients with TBI.

## 1. Introduction

Traumatic brain injury (TBI) is one of the leading causes of disability and a major public health concern worldwide [1]. Patients with TBI may present with neuropsychological symptoms, such as cognitive decline or post-traumatic stress disorders [2]. In addition, physical and sensory function impairments can also occur. These cognitive, psychological, and physical deficits were found to be sequelae of TBI, and TBI severity was associated with long-term disability [3]. After patients with acute TBI are discharged, those with cognitive and physical function impairments usually require inpatient rehabilitation programs before they are able to return to their community. However, despite intensive care and rehabilitation interventions, some patients still present with functional impairment and disability and require assistance with daily care, which cannot be provided necessarily by their previous home setting or the community. Thus, these patients with TBI who depend on others for their activities of daily living are referred to nursing homes, community-based programs specialized in TBI, and subacute facilities [4].

Although the goal of patients with TBI after inpatient rehabilitation is to return home, some are still transferred to long-term care nursing facilities. Studies investigating patients with TBI found that cognitive impairment, living alone before injury, and functional limitations are associated risk factors for institutionalization [5,6]. Other studies mentioned that older age, race, gender, geographic distribution, and acute care length of stay are associated with the risk of institutionalization [7,8,9]. In additional, Emu et al. demonstrated a model for predicting the likelihood of institutionalization after discharge from acute inpatient rehabilitation settings. They found that patients with TBI who were older, lived alone before injury, required bladder management, depended on others for transport, and were less independent in comprehension had a greater opportunity for institutionalization [5]. Although these studies identified many predictors of institutionalization after TBI, most predictors were categorical variables based on various dimensions of patients’ physical, psychological, and socioeconomic status. A holistic, comprehensive, multidimensional measurement and quantitative tool for predicting opportunity for institutionalization among patients with TBI is lacking.

In 2001, the World Health Organization (WHO) developed the International Classification of Functioning, Disability, and Health (ICF) for evaluating disability and functioning status after disease. The ICF was integrated with a biopsychosocial model of the components of body functions or structures, health conditions, limitations in daily activities, restrictions on participation, and environmental factors [10]. Based on the ICF framework, the WHO developed a holistic quantitative assessment tool for evaluating social participation, named the WHO Disability Assessment Schedule 2.0 (WHODAS 2.0). WHODAS 2.0 features the following six domains concerning functioning: cognitive, mobility, self-care, getting along with people, life activities, and social participation [11]. WHODAS 2.0 possesses multidimensional framework characteristics, and we considered it a suitable tool for analyzing the opportunity for institutionalization among patients with TBI. The aim of this study was to investigate the accuracy of WHODAS 2.0 as an objective assessment tool for predicting the opportunity for institutionalization in patients with TBI.

## 2. Methods

### 2.1. Study Design and Settings

We employed a cross-sectional study design and used a nationwide database, the Taiwan Data Bank of Persons with Disability (TDPD). We obtained the data of patients with TBI from July 2012 to December 2018. The TDPD started in July 2012 and was developed based on the ICF [12]. In Taiwan, patients with a stationary status of disability and functional impairment caused by disease can apply for disability certification. For this process, data are collected from the TDPD, and the process involves two phases of evaluation, each performed by a separate team of specialists. In the first phase of evaluation, the disease classification and ICF categories are determined by clinical physicians with related specialties, which are evaluated according to International Classification of Diseases, Ninth Revision, Clinical Modification (ICD-9-CM) and ICD-10-CM codes. Next, the physicians classify the impairment caused by the related disease according to the ICF categories of body functions (B code) and body structures (S code). The second part of the handicap application assessment is performed by people such as physiotherapists, occupational therapists, speech pathologists, psychologists, and social workers with experience in official training programs run by the Taiwan Society of ICF. They determine the impairment classification according to the participation (D code) and environmental categories (E code) of ICF and measure WHODAS 2.0 scores after participating. In this study, patients with ICD-9-CM (800–804, 850.0–850.2, 851–851.1, 852.0–853, 854.0, 900.0, and 950.0–951.5) and ICD-10-CM codes (S00, S01, S02, S03, S04, S05, S06, S07, S08, and S09) were identified as patients with TBI and retrieved from the TDPD. Other baseline variables such as age, sex, working status, education level, and urbanization level were obtained from these patients. Moreover, the severity of impairment, determined by clinical physicians on the basis of ICF categories of body functions (B codes), was obtained from the TDPD, as were the WHODAS 2.0 scores. According to the institution status in the TDPD, we subdivided our patients with TBI into two groups: community-dwelling and institutionalized groups. We compared the aforementioned variables between the groups of patients. To protect their privacy, we performed deidentification before the data analysis. This study was approved by the Institutional Review Board of Taipei Medical University (No.201804013), and informed consent was waived for the retrospective secondary data analysis.

### 2.2. WHODAS 2.0 Assessment

To assess disabilities in the TDPD, we used the traditional Chinese version of the 36-item WHODAS 2.0. As mentioned earlier, the WHODAS 2.0 has six domains with regard to functioning, each of which, together with the corresponding number of items, is outlined as follows: cognition (domain 1, six items), mobility (domain 2, five items), self-care (domain 3, four items), getting along with people (domain 4, five items), life activities (domain 5, eight items), and social participation (domain 6, eight items). Each item is rated on a five-point Likert scale (1 = “no difficulty”, 2 = “mild difficulty”, 3 = “moderate difficulty”, 4 = “severe difficulty”, and 5 = “extreme difficulty”), and the questions concern tasks performed by respondents in the past 30 days. To ensure an easier visualization of the severity of impairment, we used standardized scores from 0 to 100 in each domain and across the total 36 items; higher scores were considered to indicate greater disability in terms of performing the tasks in the WHODAS 2.0. The intraclass correlation coefficient of the traditional Chinese version of the WHODAS 2.0 was 0.80–0.89, and the internal consistency (reliability) was determined to be 0.73–0.99 (Cronbach’s α) [13,14].

### 2.3. Statistical Analyses

All continuous variables are presented as mean values, and category variables are presented as percentages. The continuous variables were compared using an independent *t*-test between groups, whereas the categorical variables were compared using a chi-square test. To identify the accuracy of the WHODAS 2.0 as a tool for assessing the opportunity for institutionalization among disabled patients with TBI, we conducted receiver operating characteristic (ROC) curve analyses on the standardized WHODAS 2.0 scores of each domain and the summary scores of all domains. The cut-off points for optimal sensitivity and specificity were determined for each domain, and the summary scores of all domains were determined using the Youden index. To analyze the possible predictors of institutionalization of disabled patients with TBI, we applied binary logistic regression to determine the opportunity for institutionalization by using baseline variables (such as gender, age, work status, family income, educational level, urbanization level, and severity of impairment) and WHODAS 2.0 standardized scores. To determine the influence of variables for predicting opportunity of institutionalization among patients with TBI, we adopted two adjusted models of analysis. Model 1 adjusted the variables of demographic data and severity of impairment, and Model 2 adjusted the demographic variables, severity of impairment, and the score of each domain of WHODAS 2.0. We used SAS 9.3 (SAS Institute, Inc., Cary, NC, USA) to perform these analyses; *p* < 0.05 was considered statistically significant.

## 3. Results

From July 2012 to October 2018, 8630 patients with TBI were enrolled in this study. Furthermore, 4895 patients with TBI (women: 32.94%) resided in the community and 3735 (women: 29.64%) were determined to be living in long-term care institutions. The independent *t*-test revealed that the institution group had higher percentages of male patients (*p* = 0.0010), older patients (*p* < 0.001), unemployed patients (*p* < 0.001), patients with lower education levels (*p* < 0.001), patients with lower family income status (*p* < 0.001), patients living in areas with lower urbanization levels (*p* = 0.0116), patients with higher severity of impairment (*p* < 0.001), and patients with higher disability scores of each domain and summary scores of all domains of WHODAS 2.0 (*p* < 0.001) (Table 1).

We found that the area under the ROC curve (AUC) was equal to 77% for the summary score of WHODAS 2.0 in predicting institutionalization of patients with TBI. Considering the sensitivity and specificity of the assessment tool in predicting institutionalization status, the summary score of WHODAS 2.0 had the highest Youden index and presented a sensitivity of 79.6% and specificity of 63.1% with a cut-off point of 66.85 in standardized summary scores. In addition to the summary score, the cognition, mobility, getting along with people, and social participation domains presented AUCs of 66% to 77% with cut-off values of the standardized score (Table 2).

In our analysis, binary logistic regression of predictors of opportunity for institutionalization revealed that older age, previous unemployment status, lower family income, higher severity of impairment, and higher WHODAS 2.0 scores in cognition, mobility, getting along, life activities, and social participation were independent contributing factors for institutionalization among patients with TBI. However, the female gender and suburban level were associated with lower opportunity for institutionalization compared with the male gender and urban level (Table 3). Taking into account the variables, we preferred Model 2 with adjusted domains of WHODAS 2.0 since it could present the independent predictors of institutionalization of TBI patients.

## 4. Discussion

TBI can lead to cognitive, emotional, and physical impairment, as well as disability. Most patients with TBI previously resided in the community before their injury. Although some patients can be discharged to their homes after intensive inpatient rehabilitation, some still need assistance that cannot be obtained from their previous living environment. To enhance the quality of life of patients with TBI and reduce the burden on their families, they must receive care in nursing homes and long-term care facilities [4]. A relevant study considered the inability to return home as a less than optimal aspect of quality of life and an economic burden on society [15]. Therefore, to effectively prepare patients with TBI for further rehabilitation after they are discharged from an acute care setting, predicting their opportunity for institutionalization is crucial. Identifying the factors related to discharge destination could aid in rehabilitation resource planning. This study found that WHODAS 2.0, as a quantitative assessment tool for predicting such patients’ institutionalization, had moderate accuracy in terms of sensitivity and specificity. Furthermore, the study revealed that older age, gender, previous unemployment status, family income, urbanization level, and severity of impairment were factors associated with institutionalization among patients with TBI.

Notably, our study results show that age was associated with institutionalization for these patients. This outcome is compatible with the findings of earlier studies that mentioned that older age can increase the risk of institutionalization among patients with TBI [5,7,16]. In addition, we found that gender was a predictor of institutionalization of disabled patients with TBI, a finding that is similar to the findings of earlier studies [17,18]. However, in contrast to a previous study that reported that women with TBI had a higher risk of institutionalization, our study showed that men had greater opportunity for entering a long-term care institution. A prospective study investigating caregiver burden indicated that more care burden was imposed on caregivers of male patients, compared with caregivers of female patients [19]. As long as male patients have more comorbidities than do female patients, the care burden would be greater in male patients with TBI, which might prompt family members to transfer them to institutions after being discharged from an acute care setting. Regarding socioeconomic status, similar to previous studies, our study revealed that the urbanization level and socioeconomic status of a family were associated with the institutionalization of patients with TBI [9]. This could be because higher family socioeconomic status would lead to a lower risk of institutionalization, which we considered to possibly be because such families can afford an in-home caregiver. Compatible with the findings of relevant studies that indicated that TBI severity was associated with institutionalization, we found that greater severity of impairment was associated with a higher opportunity for institutionalization for patients [16,20]. This indicates that a higher demand for care among disabled patients with TBI was associated with a higher risk of institutionalization.

WHODAS 2.0 can be used as a predictive assessment tool for the institutionalization of patients with TBI. Its domains of cognition, mobility, getting along with people, and social participation demonstrated moderately high accuracy in terms of discrimination (0.7 ≤ AUC < 0.8, and a lower confidence limit ≥0.7) for opportunity for institutionalization in a long-term care facility. Moreover, the standard summary score of WHODAS 2.0 had a higher AUC compared with those of the separate domains. This may show that a comprehensive and multidimensional disability status assessment can be more accurate than the use of one specific field for evaluating the possibility of institutionalization of disabled patients with TBI. This can be explained by TBI being associated with complications and clinical symptoms of varying severity caused by different etiologies. Determining the risk of requiring residency in long-term care institutions by simply using category predictors is difficult. Because of the complexity of contributing factors for institutionalization among patients with TBI, WHODAS 2.0 is a feasible assessment tool that can evaluate disability status in multiple dimensions of life and evaluate the opportunity for institutionalization.

This population-based database study revealed the association between WHODAS 2.0 and the opportunity for institutionalization among patients with TBI. It can be applied to such patients after impatient rehabilitation programs and has implications for predicting their destination after discharge. This study had several limitations that must be addressed. Firstly, the information on initial severity of injury, as well as the onset and cause of TBI, of each patient was not recorded; only the date of evaluation was recorded in the TDPD. However, according to the Regulations for the Identification of People with Disability in Taiwan, only patients with TBI for longer than six months can apply for the DES-2012 disability evaluation. In Taiwan, these patients must have been discharged from an acute rehabilitation setting based on the National Health Insurance system, which only provides inpatient rehabilitation services within six months after TBI. Furthermore, such patients must face the decision to return home or transfer to a long-term care institution. Secondly, our study could not provide the caregiver status of patients with TBI in the TDPD. Conditions such as the physical and psychological burden of family members or caregivers when caring for patients with TBI could increase the probability of these patients being institutionalized. Thirdly, bias caused by the interview process should be considered. Because some patients with TBI were cognitively impaired, some caregivers were allowed to present a proxy version of the WHODAS 2.0 questionnaire, whereas other patients with TBI could answer by themselves. Thus, inconsistency of subjectivity existed (i.e., questionnaires were either completed by the patient or caregiver), which might have led to bias in the results. Finally, on the study population, the data bank used was limited to Taiwan. Differences in social welfare status levels, races, cultures, and medical insurance systems worldwide could lead to varying outcomes on the association between WHODAS 2.0 and the opportunity for institutionalization among patients with TBI.

## 5. Conclusions

Gender, age, work status, family income, urbanization level, severity of impairment, and WHODAS 2.0 scores were the associated predictors of institutionalization in long-term care facilities for the patients with TBI. The results prove that WHODAS 2.0 can be used as a quantitative assessment tool for predicting the opportunity for long-term care facility institutionalization of patients with TBI after discharge from an acute inpatient rehabilitation setting. The summary scores of the six WHODAS 2.0 domains exhibited the highest accuracy. WHODAS 2.0 evaluation and the appropriate analysis of risk factors for institutionalization can promote efficient use of healthcare, rehabilitation, and social resources for patients with TBI.

## Figures and Tables

**Table 1:** Type of residence in relation to patients’ sociodemographic characteristics in Taiwan (*n* = 8630).

Variables	Community-Dwelling *n* = 4895		Institutionalized *n* = 3735		*p*-Value
	*n*	%	*n*	%	
Gender					0.0010
Male	3281	67.03	2628	70.36	
Female	1614	32.94	1107	29.64	
Age (years)					<0.001
18–49	1500	30.64	773	20.70	
50–64	1381	28.21	1094	29.29	
65–74	890	18.18	840	22.49	
≥75	1124	22.96	1028	27.52	
Total (mean, SD)	58.51	18.54	62.49	16.91	
Work Status					<0.001
Employed	211	4.31	28	0.75	
Unemployed	4684	95.69	3707	99.25	
Education					<0.001
≥College	122	2.49	82	2.20	
Senior high	606	12.38	470	12.58	
Junior high	1963	40.10	1236	33.09	
≤Primary	1834	37.47	1563	41.85	
No education	370	7.56	384	10.28	
Family Income Status					<0.001
Average	4797	98.00	3542	94.83	
Middle–low and low	98	2.00	193	5.17	
Urbanization Level					0.0116
Rural	794	16.22	592	15.85	
Suburban	2137	43.66	1528	40.91	
Urban	1964	40.12	1615	43.24	
Severity of Impairment					<0.001
Mild	1321	36.99	190	5.09	
Moderate	1632	33.34	596	15.96	
Severe	1042	21.29	1005	26.91	
Extreme	900	18.39	1944	52.05	
Cognition (*n*, mean ± SD) ^a^					
1-1	4866	2.08 ± 1.49	3715	3.31 ± 1.12	<0.001
1-2	4830	2.16 ± 1.42	3687	3.30 ± 1.12	<0.001
1-3	4838	2.35 ± 1.46	3702	3.45 ± 1.04	<0.001
1-4	4356	2.60 ± 1.36	3511	3.52 ± 0.92	<0.001
1-5	4891	1.70 ± 1.47	3733	3.05 ± 1.29	<0.001
1-6	4876	2.02 ± 1.54	3725	3.31 ± 1.16	<0.001
Mobility (*n*, mean ± SD) ^a^					
2-1	4831	2.52 ± 1.45	3712	3.60 ± 0.86	<0.001
2-2	4893	1.80 ± 1.57	3732	3.35 ± 1.11	<0.001
2-3	4886	1.62 ± 1.50	3590	2.98 ± 1.42	<0.001
2-4	4882	1.87 ± 1.50	3572	3.11 ± 1.34	<0.001
2-5	4745	2.67 ± 1.41	3662	3.63 ± 0.88	<0.001
WHODAS 2.0 (*n*, mean ± SD) ^b^					
Cognition (Domain 1)	4895	56.52 ± 33.01	3735	84.68 ± 24.43	<0.001
Mobility (Domain 2)	4895	56.62 ± 33.09	3735	85.79 ± 22.46	<0.001
Self-care (Domain 3)	4895	39.00 ± 34.39	3735	60.65 ± 38.13	<0.001
Getting along (Domain 4)	4895	63.28 ± 33.29	3735	88.52 ± 22.13	<0.001
Life activities (Domain 5-1)	4895	70.91 ± 36.92	3735	89.48 ± 28.03	<0.001
Social participation (Domain 6)	4895	53.37 ± 26.72	3735	72.44 ± 25.05	<0.001
Summary	4895	56.26 ± 25.79	3735	80.09 ± 18.79	<0.001

^a^ Raw Score; ^b^ percentile Score; chi-square test was used to determine proportions, and Wilcoxon rank sum test was used to determine medians. WHODAS 2.0—World Health Organization Disability Assessment Schedule 2.0.

**Table 2:** Different cut-off points along with domains for each residence status based on WHODAS 2.0 scores (*N* = 8630).

Domain	Cut-Off Point	Sensitivity (%)	Specificity (%)	Youden’s Index	AUC (95% CI)
Cognition	77.50	75.2	66.1	0.413	0.754 (0.744–0.765)
Mobility	78.13	76.0	66.2	0.422	0.767 (0.757–0.777)
Self-care	65.00	52.2	75.9	0.281	0.660 (0.648–0.672)
Getting along	87.50	76.5	64.6	0.411	0.742 (0.731–0.752)
Life activities	95.00	80.9	52.5	0.333	0.667 (0.656–0.679)
Social participation	60.42	69.2	61.4	0.307	0.701 (0.690–0.712)
Summary	66.85	79.6	63.1	0.427	0.769 (0.759–0.779)

AUC (area under the curve); CI (confidence interval; summary contains scores of six domains: cognition, mobility, self-care, getting along with people life activities, and participation in society).

**Table 3:** Logistic regression for residence status ^a^. SE—standard error.

Variables	Univariate Model				Multivariate Model 1				Multivariate Model 2			
	β	SE	Odds Ratio (OR) (95% CI)	*p*-Value	β	SE	OR (adjusted) (95% CI)	*p*-Value	β	SE	OR (adjusted) (95% CI)	*p*-Value
Gender (reference = male)												
Female	−0.16	0.047	0.86 (0.78–0.94)	0.0010	−0.25	0.054	0.78 (0.70–0.87)	<0.001	−0.22	0.056	0.80 (0.72–0.89)	<0.001
Age (ref. = 18–49)												
50–64	0.43	0.060	1.54 (1.37–1.73)	<0.001	0.43	0.069	1.54 (1.35–1.76)	<0.001	0.38	0.073	1.46 (1.27–1.68)	<0.001
65–74	0.61	0.065	1.83 (1.61–2.08)	<0.001	0.52	0.093	1.68 (1.40–2.01)	<0.001	0.41	0.098	1.50 (1.24–1.82)	<0.001
≥75	0.57	0.062	1.78 (1.57–2.00)	<0.001	0.40	0.092	1.49 (1.25–1.79)	<0.001	0.20	0.096	1.23 (1.02–1.48)	0.0337
Work Status (reference = employment)												
Unemployed	1.79	0.202	5.96 (4.01–8.87)	<0.001	1.01	0.221	2.73 (1.77–4.22)	<0.001	0.76	0.239	2.15 (1.35–3.43)	0.0014
Education (reference = ≥college)												
Senior high	0.14	0.156	1.15 (0.85–1.57)	0.3572	0.23	0.175	1.26 (0.89–1.77)	0.1935	0.18	0.183	1.19 (0.83–1.71)	0.3335
Junior high	−0.07	0.147	0.94 (0.70–1.25)	0.6577	0.14	0.166	1.15 (0.83–1.59)	0.4025	0.18	0.174	1.20 (0.85–1.68)	0.3015
≤Primary	0.24	0.147	1.27 (0.95–1.69)	0.1060	0.12	0.171	1.13 (0.81–1.58)	0.4667	0.11	0.178	1.12 (0.79–1.59)	0.5262
Illiterate	0.43	0.160	1.54 (1.13–2.11)	0.0067	0.23	0.187	1.25 (0.87–1.81)	0.2279	0.23	0.195	1.26 (0.86–1.85)	0.2362
Family Income (reference = average)												
Middle–low and low	0.98	0.126	2.67 (2.08–3.41)	<0.001	0.81	0.139	2.24 (1.70–2.94)	<0.001	0.79	0.144	2.20 (1.66–2.92)	<0.001
Urbanization level (reference = urban)												
Rural	−0.10	0.064	0.91 (0.80–1.03)	0.1251	−0.16	0.072	0.85 (0.74–0.98)	0.0251	−0.11	0.075	0.90 (0.78–1.04)	0.1564
Suburban	−0.14	0.047	0.87 (0.79–0.95)	0.0032	−0.18	0.053	0.84 (0.76–0.93)	<0.001	−0.18	0.056	0.83 (0.75–0.93)	0.0010
Severity of impairment (reference = mild)												
Moderate	0.93	0.091	2.54 (2.12–3.04)	<0.001	0.85	0.092	2.34 (1.95–0.80)	<0.001	0.60	0.097	1.81 (1.50–2.19)	<0.001
Severe	1.90	0.089	6.71 (5.63–7.99)	<0.001	1.79	0.091	6.02 (5.04–7.19)	<0.001	1.13	0.097	3.09 (2.55–3.74)	<0.001
Extreme	2.71	0.087	15.02(12.65–17.83)	<0.001	2.61	0.089	13.63 (11.45–16.21)	<0.001	1.52	0.100	4.58 (3.76–5.57)	<0.001
Domain Score ^b^												
Cognition	1.78	0.048	5.90 (5.37–6.49)	<0.001					0.34	0.079	1.40 (1.20–1.63)	<0.001
Mobility	1.82	0.049	6.19 (5.62–6.81)	<0.001					0.72	0.070	2.06 (1.80–2.36)	<0.001
Self-care	1.24	0.047	3.44 (3.14–3.77)	<0.001					−0.02	0.063	0.98 (0.87–1.11)	0.7712
Getting along	1.78	0.049	5.94 (5.40–6.53)	<0.001					0.34	0.079	1.40 (1.20–1.64)	<0.001
Life activities	1.54	0.051	4.67 (4.23–5.16)	<0.001					0.39	0.065	1.48 (1.31–1.68)	<0.001
Social participation	1.28	0.046	3.59 (3.28–3.92)	<0.001					0.27	0.061	1.31 (1.16–1.48)	<0.001

^a^ Residence status: event is defined as institution; ^b^ domain score cut by cut point score (if WHODAS score ≥ cut point, then domain score = 1; otherwise, domain score = 0).
